# Adsorption Properties for La(III), Ce(III), and Y(III) with Poly(6-acryloylamino-hexyl hydroxamic acid) Resin

**DOI:** 10.3390/polym13010003

**Published:** 2020-12-22

**Authors:** Xiaoyan Cao, Chunjie Zhou, Shuai Wang, Ruilin Man

**Affiliations:** 1 College of Chemistry and Chemical Engineering, Hunan Provincial Key Laboratory of Efficient and Clean Utilization of Manganese Resources, Central South University, Changsha 410083, China; caoxxyu@126.com (X.C.); zhouchunjietc@163.com (C.Z.); rlman@csu.edu.cn (R.M.); 2 College of Chemistry and Chemical Engineering and Jiangxi Province Engineering Research Center of Ecological Chemical Industry, Jiujiang University, Jiujiang 332005, China

**Keywords:** adsorption, hydroxamic acid resin, rare earth ions, synthesis

## Abstract

Using polyacrylic resin followed by the substitution reaction with 6-aminohexyl hydroxamic acid, poly(6-acryloylamino-hexyl hydroxamic acid) resin (PAMHA) was successfully synthesized. PAMHA, a spherical resin with the particle size of 0.4 mm, is a novel polyamide hydroxamic acid chelating resin containing acylamino and hydroxamic acid functional groups. A series of influences (pH, contact time, temperature, and the initial concentrations of rare earth ions) were investigated to determine the adsorption properties. The adsorption capacity for La(III), Ce(III), and Y(III) ions were 1.030, 0.962, and 1.450 mmol·g^−1^, respectively. Thermodynamic and kinetic studies were also carried out to show that the uptake of rare earth ions onto PAMHA fitted well the pseudo-second-order model and Langmuir isotherm, and the adsorption process was spontaneous endothermic. In addition, desorption of rare earth ions was achieved by using 2 mol·L^−1^ HNO_3_ and desorption efficiencies for La(III), Ce(III), and Y(III) ions were 98.4, 99.1, and 98.8%, respectively. Properties of PAMHA resin were characterized by scanning electron microscope (SEM), Fourier transform infrared spectrometry (FTIR), and X-ray photoelectron spectrometer (XPS). The results showed that there was coordination between the rare earth ions with PAMHA and rare metal ions were chemically adsorbed on the surface of the PAMHA.

## 1. Introduction

Ion exchange and adsorption technology is an important chemical separation method for high efficiency extraction, concentration, and purification. Since the 1930s, ion exchange and adsorption technology has developed rapidly with the invention of ion-exchange resin synthesis. Ion exchange and adsorption resin is a type of functional polymer material with network structure. It is widely used in light industry [1,2], food [3,4], medicine [5,6], environmental protection [7,8], and hydrometallurgy [9,10,11]. Chelating resin, as a special ion exchange resin having chelating functional groups, has the advantage of high selection during adsorption process and easy regeneration during desorption process [10]. Because of its functional groups containing O, N, S, and P atoms with lone pair electrons, chelating resin can grasp metal ions and form a chelated structure [12]. Therefore, by appropriate selection of functional groups, resins can be designed to coordinate with a variety of metal ions.

Hydroxamic acid can be regarded as a derivative of carboxylic acid and exhibit a strong ability to chelate metal ions. Due to its unique structure, hydroxamic acid can chelate two or three hydroxamate molecules to form five-membered ring complexes, which are widely used as analytical reagents [13], drugs [14], flotation collectors [15,16], and chelating resins [17,18]. 

Polymeric chelating resins containing the hydroxamic acid group can form complexes with transition metal ions. Hydroxamic acid polymers [19] contain both carbonyl and oxime groups (RC (=O) NHOH) in which oxime group has position of oxygen and nitrogen coming together, and these two atoms have lone pair electrons. Consequently, hydroxamic acid polymers exhibit strong chelating ability to metal ions. The research of polymeric hydroxamic acid began in 1954 when Cornaz et al. [20,21] prepared hydroxamic acid derivatives of Amberlite IRC-50. Since then, much attention had been given to the synthesis of poly(hydroxamic) acid chelating resins and application. In the 1980s, Vernon et al. [22,23] prepared a poly-hydroxamic acid ion exchanger and studied its application to the separation of several transition metal species. Agrawal et al. [24] synthesized six poly(styrene-p-hydroxamic acids) and these polymers were used as chelating ion-exchange resins for the separation and determination of rare earths. Haron et al. [25] investigated using tin-loaded poly(hydroxamic acid) chelating resin for removal of arsenate ions from aqueous solution. In recent years, some researchers have paid attention to the study of novel hydroxamic acid chelating resin. Polyhydroxamic acid (PHA) cartridge had been developed for the preconcentration of rare earth elements (REEs) and separation of the matrix components in seawater by Kumar et al. [26], and the synthetic resin was found to have good selectivity for rare earth elements. Cheng et al. [27] researched the adsorption of Cu(II) onto polyvinyl alcohol-methacrylate-hydroxylamine (PVA-MA-HH) resin. The team of Rahman applied new poly(hydroxamic acid) chelating ligand from the khaya cellulose-graft-poly(methyl acrylate) (PMA) for transition metal ions removal from aqueous media [28]. Jiao et al. [29] reported a poly(amidoxime-hydroxamic acid) (PAHA) cellulose derivative and studied the adsorption properties of PAHA cellulose for heavy metal ion removal.

The rare earth metals consist of a group of 17 chemical elements, including a series of lanthanides, scandium (Sc), and yttrium (Y) [30]. Owing to their similar chemical and physical properties, it is very difficult to separate rare earth metals using common chemical methods. The method of adsorption is considered a promising alternative to conventional processes by its simplicity, low cost, high efficiency, and wide availability for the enrichment and separation of rare earth elements. The key to adsorption separation technology is the development of adsorption separation materials. Hydroxamic acid chelating resins can provide electron pairs to bind forces for rare earth metals, hence, introducing hydroxamic acid resins to the enrichment and separation of rare earth elements has some potential advantages.

In our previous research [31,32], we synthesized a series of poly(hydroxamic acid) chelating resins. We also investigated the adsorption to rare earth ions and found that the synthesized resins had good adsorption capacities for La(III), Ce(III), and Y(III) ions. The preparation and application of polymers bearing acylamino groups and hydroxamic acid functional groups has not been reported. Therefore, as a continual work, we design another novel chelating resin (poly(6-acryloylamino-hexyl hydroxamic acid) resin—PAMHA) by grafting 6-aminohexyl hydroxamic acid onto polyacrylic resin (D113). The adsorption performances to La(III), Ce(III), and Y(III) ions with the synthetic resin were investigated. The kinetics, isotherms, and thermodynamics of adsorption of La(III), Ce(III), and Y(III) ions on PAMHA were systematically investigated. The influence factors of pH solution, initial concentration, contact time, and temperature were discussed. Meanwhile, PAMHA was characterized by scanning electron microscopy (SEM), X-ray photoelectron spectrometer (XPS), and Fourier transform infrared spectroscopy (FTIR) to clarify the adsorption mechanism of rare earth ions onto it.

## 2. Experimental and Methods

### 2.1. Quantum Chemical Calculation of Poly(6-acryloylamino-hexyl hydroxamic acid) Resin (PAMHA)

Firstly, the initial molecular structure of acrylamide and acrylamide hexyl hydroxamic acid was established by ChemOffice 2019. Then, MM2 and PM3 methods were used for preliminary optimization. At last, the B3LYP method and 6-31G(d) group in the Gaussian 03 were employed to optimize the molecular configuration, the quantum chemical parameters were calculated.

### 2.2. Materials and Apparatus

In the experiment, caprolactam, hydroxylamine hydrochloride, sodium hydroxide, and toluene were of analytical grade. D113 resin was purchased from Shanghai Huizhu resin Co., Ltd., Shanghai, China. Required stock standard solution of REEs were prepared by dissolving La(NO_3_)_3_·6H_2_O, Ce(NO_3_)_3_·6H_2_O, and Y(NO_3_)_3_·6H_2_O in distilled water, respectively. 

Adsorption experiments were carried out in the SHA-C thermostatic vibrator (Changzhou Aohua Instrument Co., Ltd., Changzhou, China). The Fourier transform infrared spectra (FTIR) (G510FTIR, Nicolet, Madison, WI, USA) was performed to investigate the skeleton vibration. The morphology surface of the resin was determined by scanning electron microscopy (SEM) (Mira3, Tescan, Brno, Czech Republic). X-ray photoelectron spectrometer (XPS) (Escalab250Xi, Thermo Fisher, Waltham, MA, USA) was applied to the surface composition of the resin.

### 2.3. Preparation of 6-aminohexyl hydroxamic acid

**Scheme 1:**
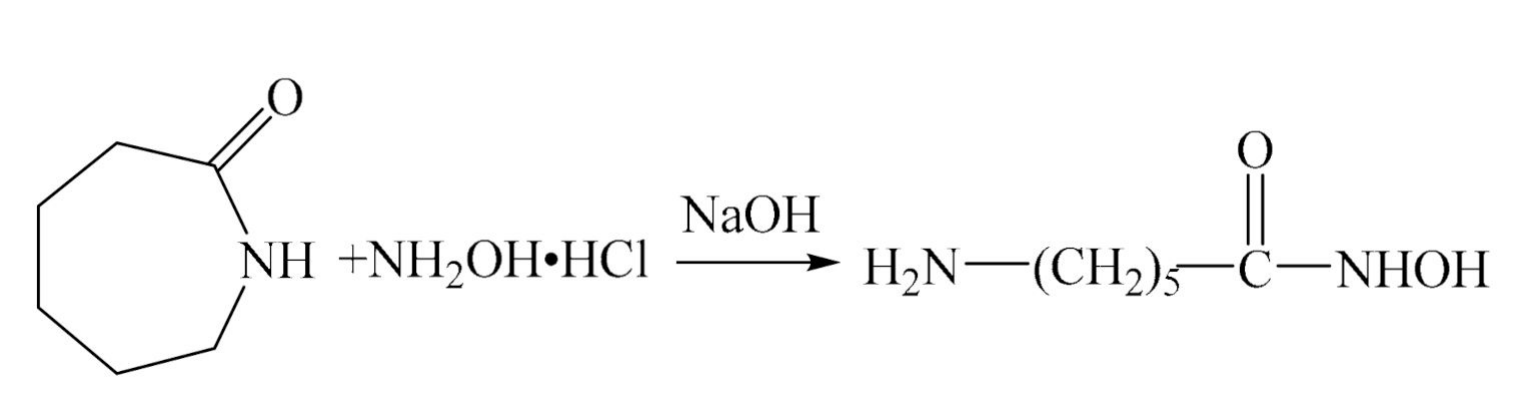
Synthesis of 6-aminohexyl hydroxamic acid.

The synthetic route of 6-aminohexyl hydroxamic acid was presented in Scheme 1. Taking toluene as a solvent, caprolactam (11.3 g), hydroxylamine hydrochloride (6.9 g), and sodium hydroxide (4.0 g) were added into a 250 mL three-necked flask equipped with a magnetic stirrer. After reaction for 2 h at 100 °C, solution was obtained by acidification, filtration, and rotary evaporation.

### 2.4. Preparation of PAMHA 

**Scheme 2:**
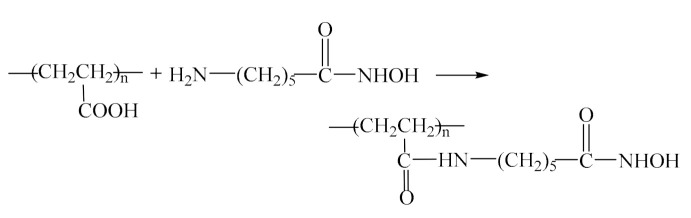
Synthesis route of poly(6-acryloylamino-hexyl hydroxamic acid) resin (PAMHA).

The synthetic route of PAMHA is shown in Scheme 2. A certain proportion of D113 resin and 6-aminohexyl hydroxamic acid were placed into a 250 mL three-necked flask, which was equipped with a mechanical stirrer and condenser, and immersed in a thermostatic oil bath. After reaction under the certain conditions (molar ration of carboxyl to amine 1:1, reaction time 2 h, reaction temperature 100 °C), the solid in the reactive mixture was filtered, washed several times with distilled water and ethanol. Finally, a pale orange granular PAMHA resin was synthesized accordingly (yield: 67.07%).

### 2.5. The Adsorption and Desorption Experiments

Adsorption was performed out by the static method with different pH, concentrations, contact time, and temperature of solutions. In the experiment, 0.1 g of dry resin was added to 50 mL of rare earth ions solutions of initial concentration 0.01 mol·L^−1^. Then, the contents were placed in the temperature-controlled water bath shaker (SHA-C) with the shaking rate setting as 200 rpm for oscillation appropriate time. Finally, the supernatant liquids were filtered, and the metal concentration was measured by an ultraviolet (UV) spectrophotometer. The adsorption capacity of the resin was calculated by Equation (1).

(1) $$Q=\frac{(C_{0}-C_{t})V}{m}$$

where *Q* (mmol·g^−1^) is the adsorption capacity; *C*_0_ (mol·L^−1^) and *C_t_* (mol·L^−1^) are the initial and concentrations of rare earth metal ions at *t* time, respectively; *V* (L) is the volume of experiment solution, and *m* (g) is the mass of dry resin.

After adsorption, REEs ions-loaded PAMHA were collected and gently washed with distilled water, then contacted with 50 mL of 2 mol·L^−1^ HNO_3_ as eluting agent. Under the conditions of shaking 5 h at 30 °C, the mixture was filtered and the rare earth ions in the aqueous solution were similarly determined as described above. The desorption efficiency of REEs ions from PAMHA was calculated according to the formula in Equation (2). In order to evaluate the regeneration of PAMHA resin, the adsorption–desorption operation was repeated in six cycles.

(2) $$R_{W}=\frac{C_{W}V_{W}}{Qm}\times 100\%$$

where *R_w_* (%) is the desorption efficiency; *C_w_* (mol·L^−1^) is the concentrations of rare earth metal ions in eluent solutions; *V_w_* (L) is the volume of eluent; *Q* (mmol·g^−1^) is the adsorption capacity, and *m* (g) is the mass of dry resin.

### 2.6. Determination of Metal Ion Concentration

The concentrations of rare earth ions were determined by spectrophotometry. In each experiment, we used arsenazo as a color developer to form a blue complex with La(III) ion, tribromoasenazo with Ce(III) ion, and arsenazo with Y(III) ion. Then the La(III), Ce(III), and Y(III) ions concentration were calculated using the UV absorbance at the wavelength of 656 nm, 635 nm, and 651 nm, respectively.

## 3. Results and Discussions

### 3.1. Quantum Chemical Calculation 

**Figure 1:**
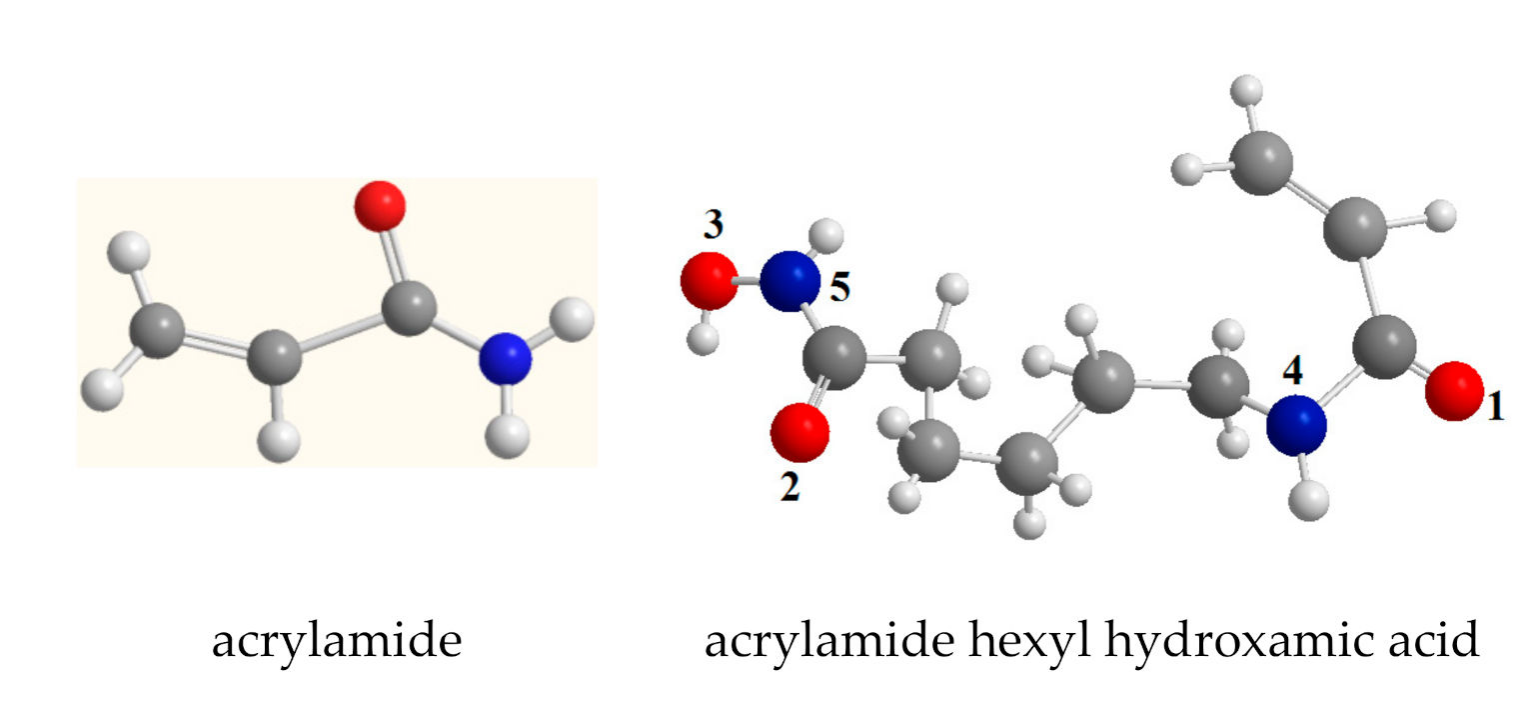
Optimization of molecular structure.

Figure 1 shows the optimization of molecular structure. The calculation results of molecular energy, the highest occupied molecular orbital (HOMO) energy, and the lowest unoccupied molecular orbital (LUMO) energy are listed in Table 1. Table 2 shows the net charges of selected atoms on acrylamide hexyl hydroxamic acid.

It can be seen that the HOMO (highest occupied molecular orbital energy) value of acrylamide hexyl hydroxamic acid was larger than that of acrylamide and the LUMO (lowest unoccupied molecular orbital energy) of acrylamide hexyl hydroxamic acid was less than that of acrylamide. The HOMO value of the molecule reflects the strength of the electron-donating ability. The higher the HOMO is, the stronger the electron donating ability is. However, LUMO energy presents the ability of receiving feedback electrons to form π bond, smaller and easier to receive the feedback π electrons provided by transition metals, thus to improve the strength and selectivity of coordination bond.

According to Table 2, we can conclude that the negative charges were distributed on nitrogen and oxygen atoms. It indicated that nitrogen and oxygen atoms were the active sites of the reaction between the molecule and metal ions.

Therefore, we can draw a conclusion that PAMHA resin is expected to have good coordination performance for rare earth ions, and nitrogen and oxygen atoms of the resin are the adsorption active points.

### 3.2. Characterization

#### 3.2.1. Scanning Electron Microscopy (SEM) 

**Figure 2:**
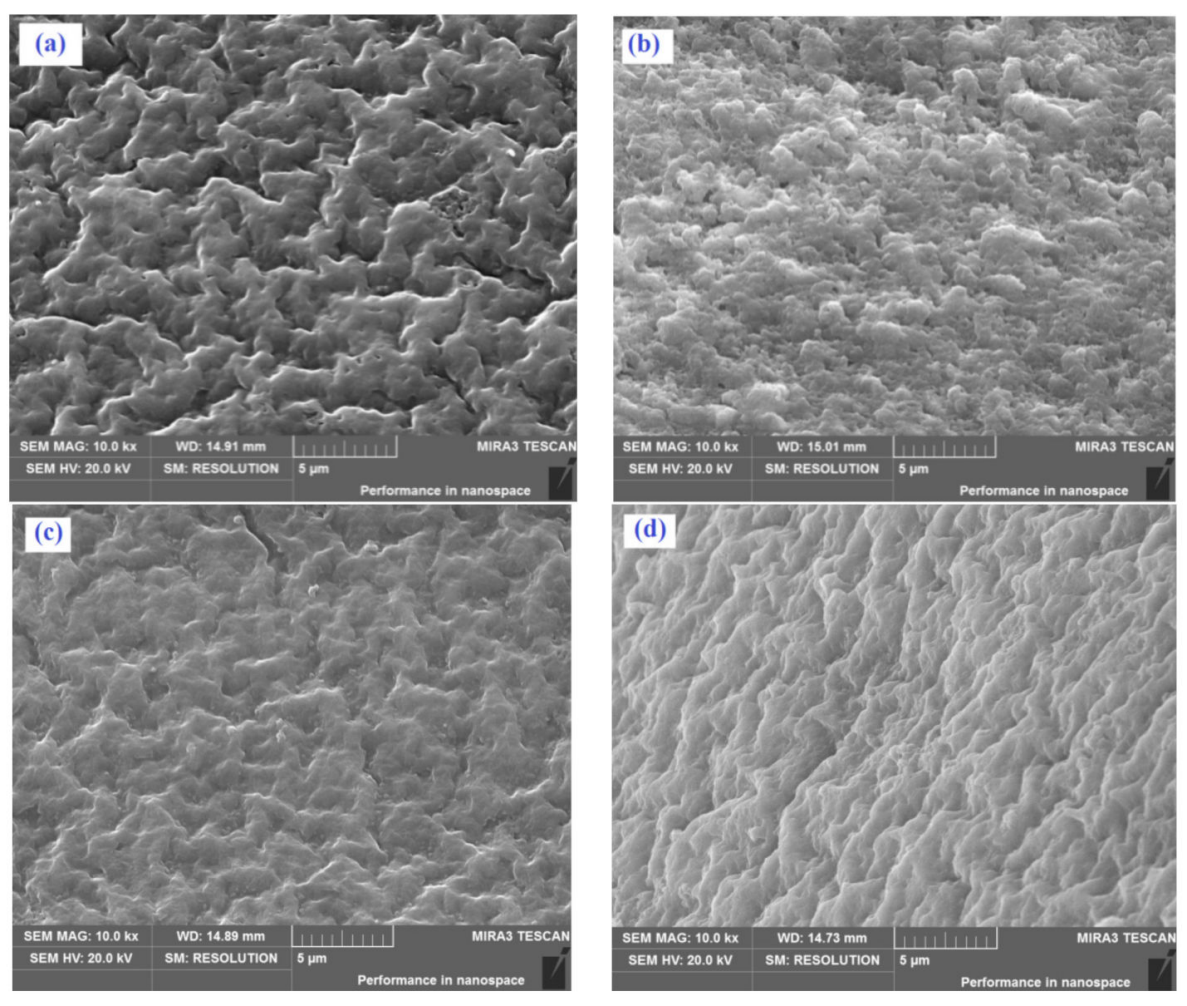
Scanning electron microscope (SEM) photographs of PAMHA (**a**) before, and after (**b**) La(III), (**c**) Ce(III), or (**d**) Y(III) adsorption.

The SEM analysis is an efficient way to observe the morphology and the images of PAMHA. La(III)-loaded PAMHA, Ce(III)-loaded PAMHA, and Y(III)-loaded PAMHA are displayed in Figure 2. It can be seen from Figure 2a that the surface of PAMHA was rough and porous, which is beneficial to preserving its large specific area and providing additional channels for adsorption of rare earth ions. After adsorption of La(III), Ce(III), and Y(III), the surface of PAMHA was covered with a layer of adsorbed metal ions, which suggests that these three ions were adsorbed on the surface of PAMHA.

#### 3.2.2. Fourier Transform Infrared Spectroscopy (FTIR) 

**Figure 3:**
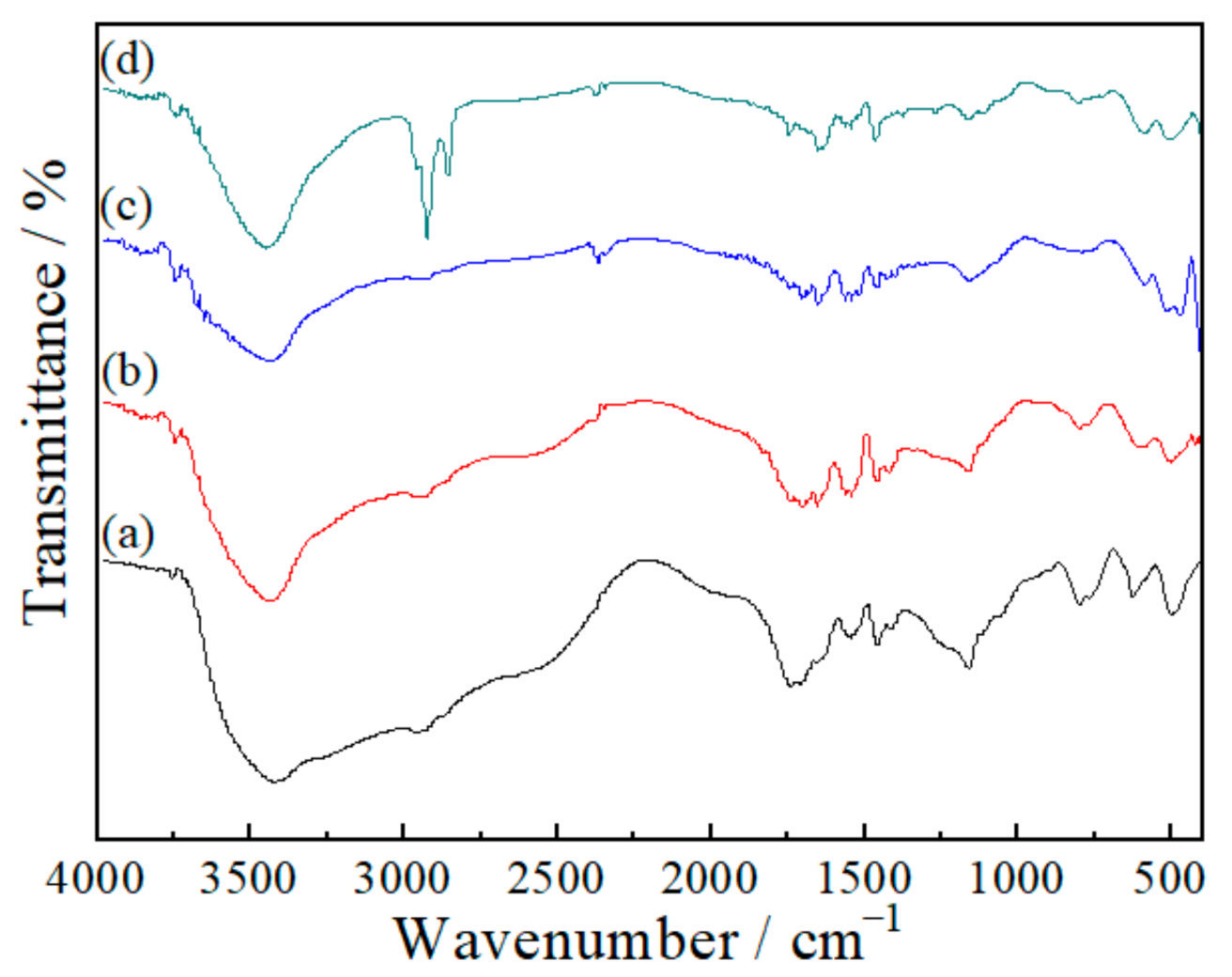
FTIR of PAMHA (**a**) before, and after (**b**) La(III), (**c**) Ce(III), or (**d**) Y(III) adsorption.

In the experiment, KBr method was used to determinate infrared spectrum of resin on G510PFTIR infrared spectrometer. Figure 3 and Table 3 show the FTIR spectra of PAMHA before and after treatment by La(III), Ce(III), and Y(III). Analysis of infrared spectra, the characteristic peak of hydroxamic acid appeared at 3419 cm^−1^, and it can be assigned to N–H and O–H superposition peak of stretching vibrations [33]. The peak at 1741 cm^−1^ was assigned to C=O stretching vibration [34]. However, after the PAMHA resin was treated by La(III), Ce(III), or Y(III) solution, the peaks of N–H and O–H superposition shifted to a higher frequency of around 3446 cm^−1^ and the peak of C=O moved to a lower one about 1701 cm^−1^. These results revealed that there was coordination between the rare earth ions with PAMHA and rare mental ions were chemically adsorbed on the surface of the PAMHA. The relevant explanation of a shift in the spectra is that there is an effect of metal adsorption on the functional groups and the metal-ligand chelate ring was formed [35].

#### 3.2.3. X-ray Photoelectron Spectroscopy (XPS) 

**Figure 4:**
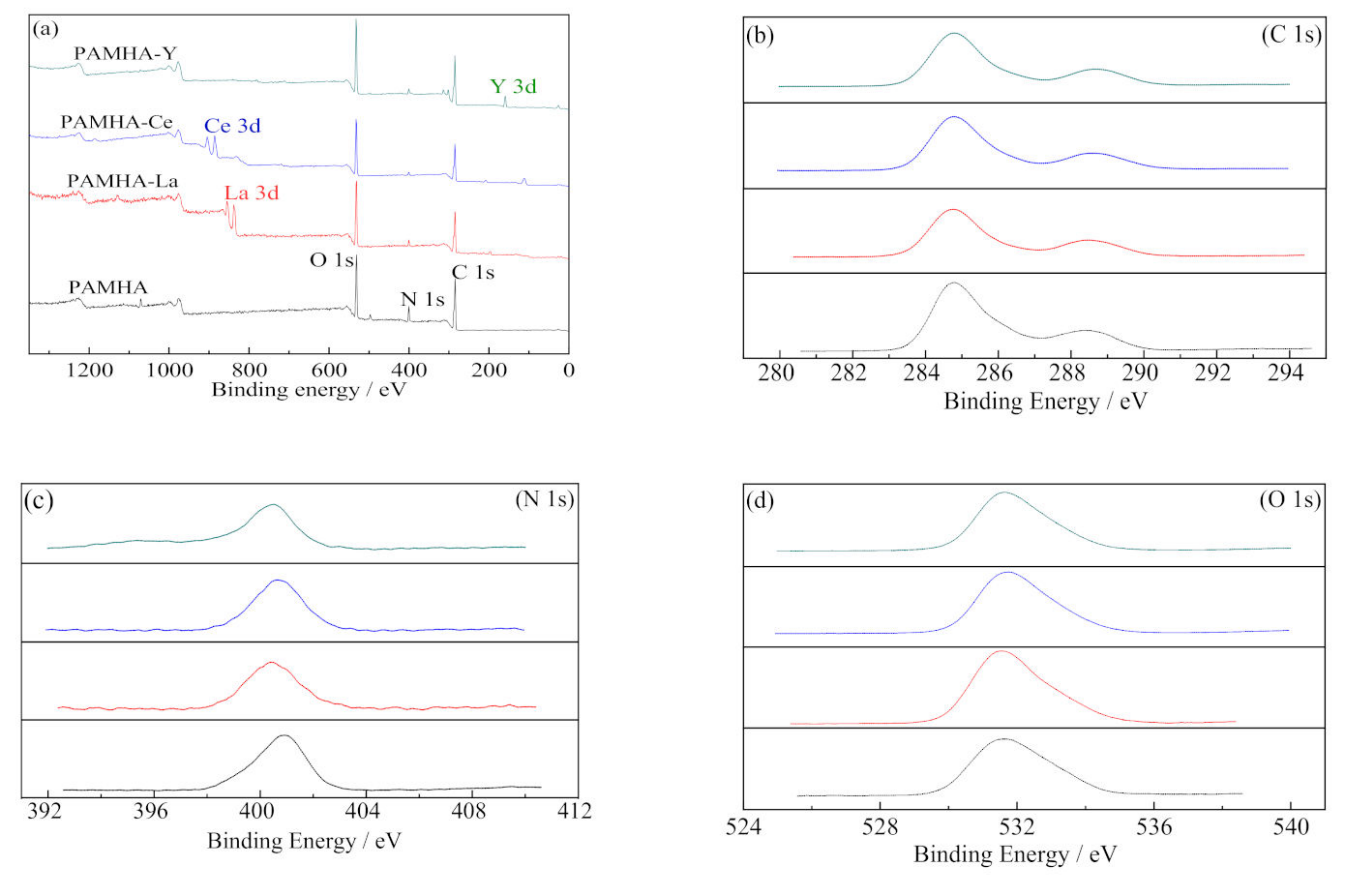
X-ray photoelectron spectroscopy (XPS) spectra of PAMHA before and after adsorption. (**a**) Full spectrum; (**b**–**d**) high-resolution XPS spectra of C 1s, N 1s, and O 1s.

The survey scan XPS spectra of PAMHA before and after treatment by La(III), Ce(III), and Y(III) over a binding energy range of 0–1200 eV were shown in Figure 4. The electronic binding energy of elements C 1s, N 1s, O 1s and La 3d, Ce 3d, Y 3d determined by XPS are listed in Table 4 and then compared with the spectrum before adsorption. New signals of La 3d, Ce 3d, and Y 3d after adsorption demonstrated the presence of La(III), Ce(III), and Y(III) cations in the adsorbed PAMHA. Moreover, binding energy of elements O 1s increased from 531.98 eV to 532.03 eV and N 1s decreased from 400.87 eV to 400.32 eV, which indicated a complexation reaction between N, O, and metal ions.

### 3.3. Adsorption Capacity of PAMHA on Rare Earth Ions

Adsorption capacity is an important parameter to evaluate the performance of adsorption resin. Under the conditions of pH 2, contact time 12 h, temperature 45 °C, and initial concentration 0.01 mol·L^−1^, the adsorption capacity of rare earths ions on D113 and PAMHA were comparatively measured (see Table 5). It is appeared that the sorption of PAMHA for La(III), Ce(III), and Y(III) was significantly larger than that of D113. The result can be explained that D113 grafted hydroxyl and oxime groups had strong adsorption capacity for rare earth metal ions and the adsorption capacity of PAMHA had been increased accordingly.

### 3.4. Effect of Solution pH on Adsorption

**Figure 5:**
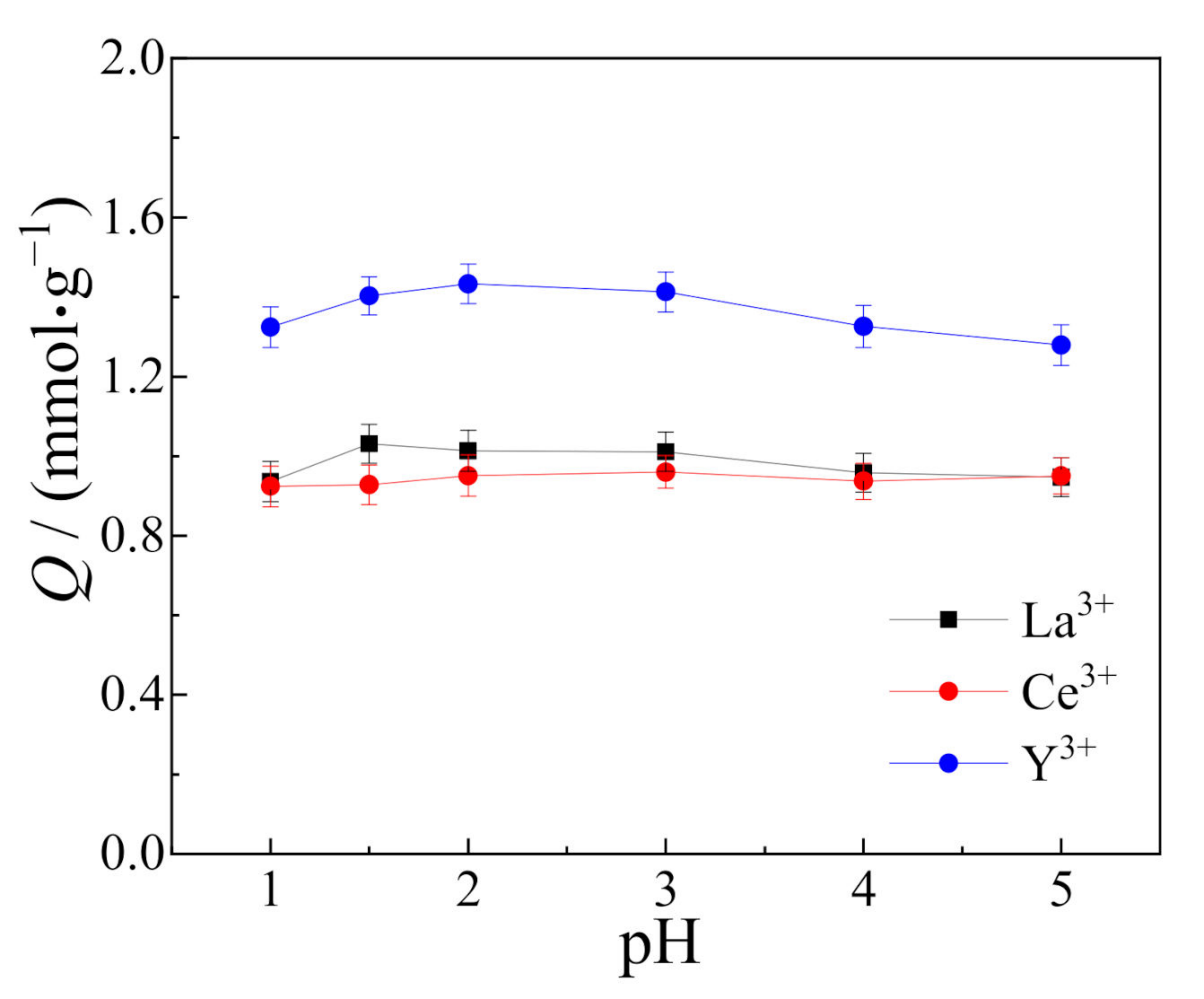
Solution pH effect on the adsorption capacity of PAMHA.

To investigate the effect of solution pH on the adsorbability of PAMHA for rare earth ions, the static adsorption experiments were conducted at initial concentration of rare earth ions 0.01 mol·L^−1^, 45 °C and 5 h, and the pH varied from 1.0 to 5.0. The results shown in Figure 5 noted that the adsorption capacity for La(III), Ce(III), and Y(III) ions showed increase–decrease trend. The maximum adsorptions for La^3+^, Ce^3+^, and Y^3+^ were 1.031, 0.96, and 1.433 mmol·g^−1^, when the optimal pH values for La^3+^, Ce^3+^, and Y^3+^ were 1.5, 3.0, and 2.0, respectively.

### 3.5. Kinetic Adsorption

**Figure 6:**
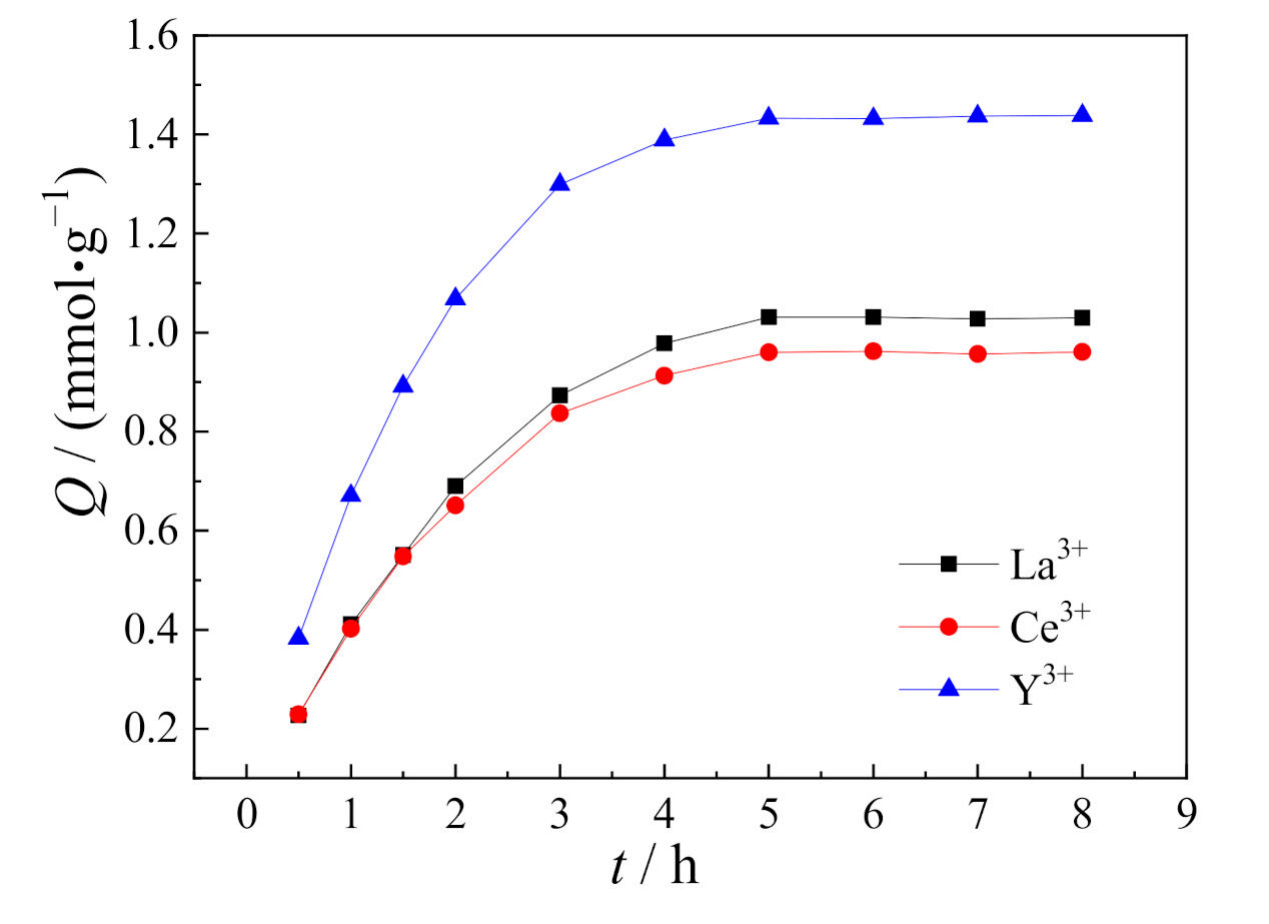
Effect of contact time on the adsorption capacity of PAMHA. (*C*_0_ = 0.01 mol·L^−1^, 45 °C, pH for La^3+^, Ce^3+^, and Y^3+^ were 1.5, 3.0, and 2.0, respectively.).

Figure 6 reflected the effect of contact time on the adsorption capacity of PAMHA at 45 °C. As seen in Figure 6, the adsorption capacities of rare earth ions increased significantly in the first 3 h; this is because, initially, the adsorption sites for rare earth ions on PAMHA were sufficient [36].With the increase of time, more and more adsorption sites were occupied and saturating adsorption capacity was reached eventually. On the basis of the kinetic results, it was considered appropriate that the equilibrium times for La^3+^, Ce^3+^, and Y^3+^ were 5 h, 6 h, and 6 h, respectively, in the following experiments.

For the sake of better understanding the adsorption process, the dynamical experimental data was analyzed by different models, including pseudo-first-order model, pseudo-second-order model, and Elovich model [37,38,39,40]. The models are respectively described in the following Equations.

(3) $$\log\left(Q_{e}-Q_{t}\right)=\log Q_{1}-\frac{k_{1}}{2.303}t$$

(4) $$\frac{t}{Q_{t}}=\frac{1}{k_{2}Q_{2}{}^{2}}+\frac{t}{Q_{2}}$$

(5) $$Q_{t}=\frac{1}{\beta }\ln(\alpha \beta )+\frac{1}{\beta }\ln t$$

where *Q*_e_ (mmol·g^−1^) and *Q_t_* (mmol·g^−1^) are adsorption capacities at equilibrium and at time *t*, *Q*_1_ (mmol·g^−1^) and *Q*_2_ (mmol·g^−1^) are the calculated adsorption capacities of the pseudo-first-order model and pseudo-second-order model, separately; *k*_1_ (h^−1^) and *k*_2_ (g·mmol^−1^·h^−1^) are the first order constant and the second order constant, *α* (g·mmol^−1^·h^−1^) is the initial adsorption rate, and *β* (g·mmol^−1^) is an indicator of the number of positions possible for the adsorption.

**Figure 7:**
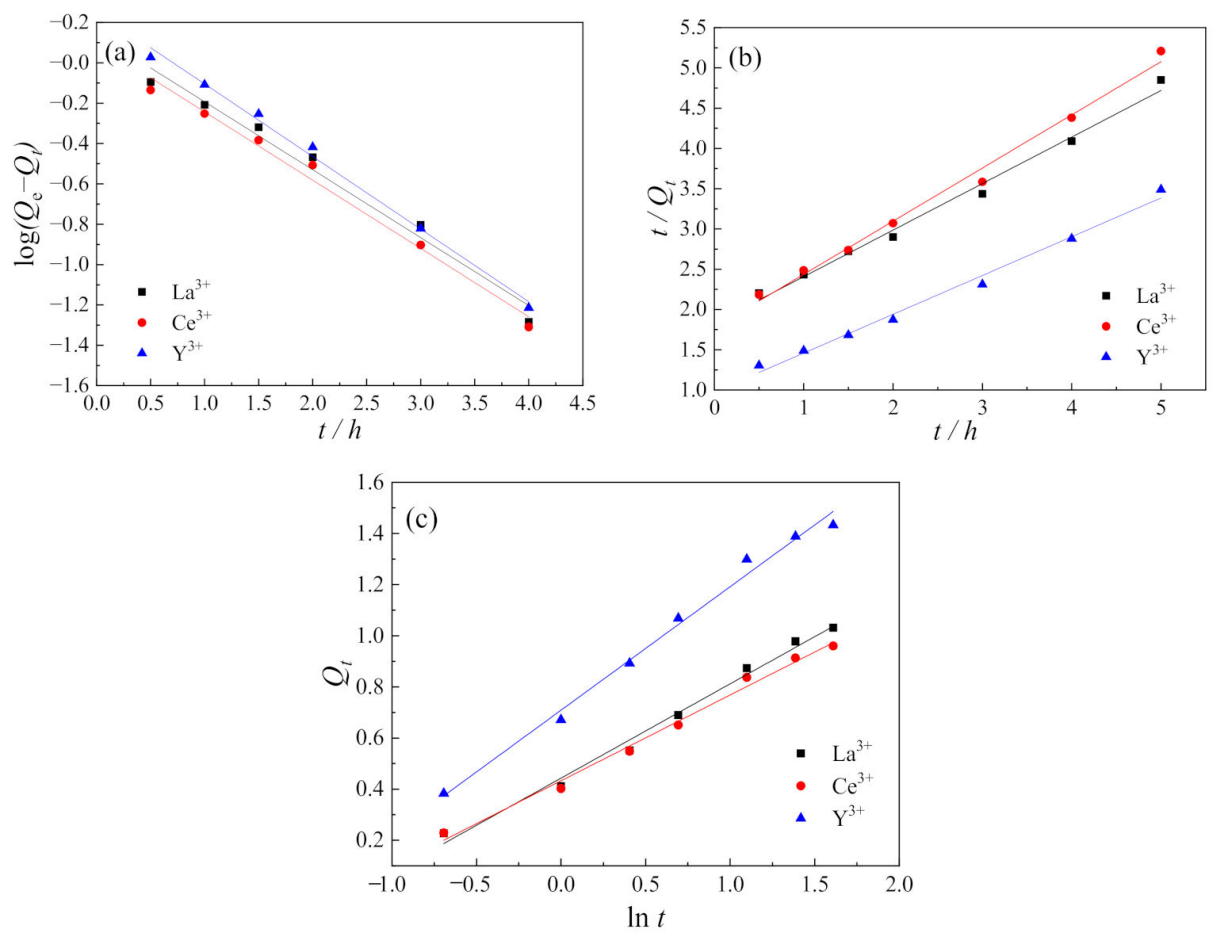
Linear plots of different kinetic models: (**a**) Pseudo-first-order model; (**b**) pseudo-second-order model; (**c**) Elovich equation.

Figure 7 showed the linear plots of log *(Q*_e_ − *Q_t_)* vs. *t*, *t*/*Q_t_* vs. *t*, and *Q_t_* vs. ln*t*. Meanwhile, the related kinetic parameters are listed in Table 6. The experimental result indicates that, compared with the other two models, pseudo-second-order model was more suitable for characterizing the kinetic data, the interaction of rare earth ions with PAMHA resin followed the pseudo-second-order kinetics. Therefore, the adsorption process can be described by liquid membrane diffusion and chemical reaction, suggesting that rare earth ions might chemisorb onto the PAMHA surface [12].

### 3.6. Isotherm Adsorption

**Figure 8:**
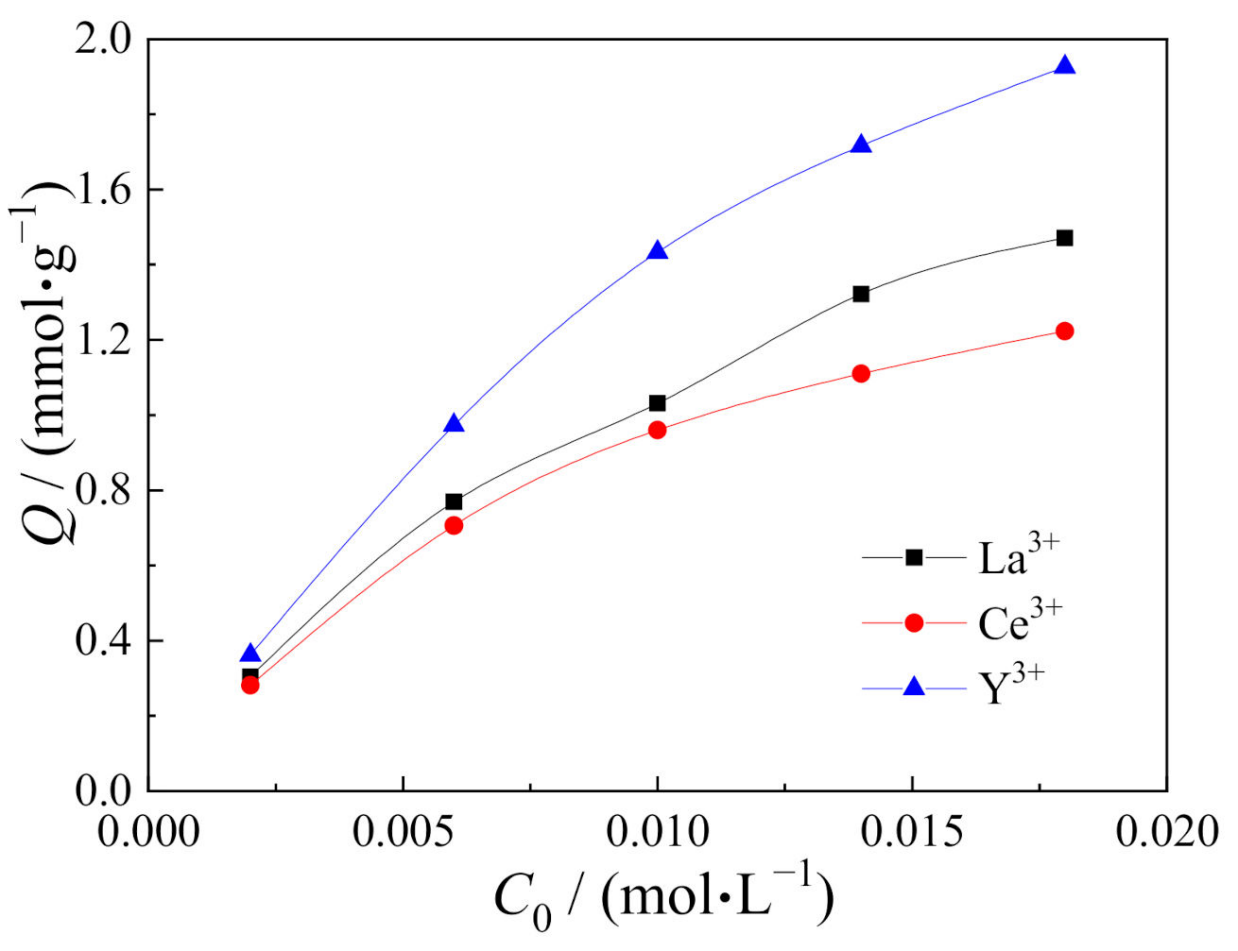
Effect of initial concentration of metal ions on the adsorption capacity of PAMHA (pH for La^3+^, Ce^3+^, and Y^3+^ were 1.5, 3.0, and 2.0, respectively, 5 h, 45 °C).

The influence of the initial concentration of metal ions on PAMHA adsorption was investigated as the concentration of metal ions was evaluated in the range of 0.002–0.018 mol·L^−1^. The results are displayed in Figure 8. As shown in Figure 8, the adsorption capacity increased with the increase of metal ions concentration. This phenomenon indicated that the increase of the initial concentration can lead to the increase of driving force of the adsorption process [41,42], thus it can improve the contact opportunities between metal ions and the effective active sites in the solution.

Adsorption isotherm models can provide mechanism information of the adsorption process [43]. The adsorption isotherm reflects that, at the same temperature, the adsorption capacity changes with the initial concentration of ions regularly. In the experiment, the commonly used isothermal adsorption models, such as Langmuir and Freundlich isotherms, were engaged to depict the adsorption equilibrium rare earth ions onto the PAMHA resin.

The linear form of the Langmuir isotherm is expressed by the following Equation [44].

(6) $$\frac{C_{e}}{Q_{e}}=\frac{C_{e}}{Q_{m}}+\frac{1}{Q_{m}K_{L}}$$

The linear form of the Freundlich isotherm is given by the following Equation [45].

(7) $$\ln Q_{e}=\frac{1}{n}\ln C_{e}+\ln K_{F}$$

where *Q*_e_ (mmol·g^−1^) is adsorption capacity at equilibrium, *Q*_m_ (mmol·g^−1^) is the maximum adsorption capacity of Langmuir, *C*_e_ (mol·L^−1^) is the equilibrium concentration, *K*_L_ (L·mol^−1^) is Langmuir constant, *n* and *K*_F_ are Freundlich adsorption coefficients. 

**Figure 9:**
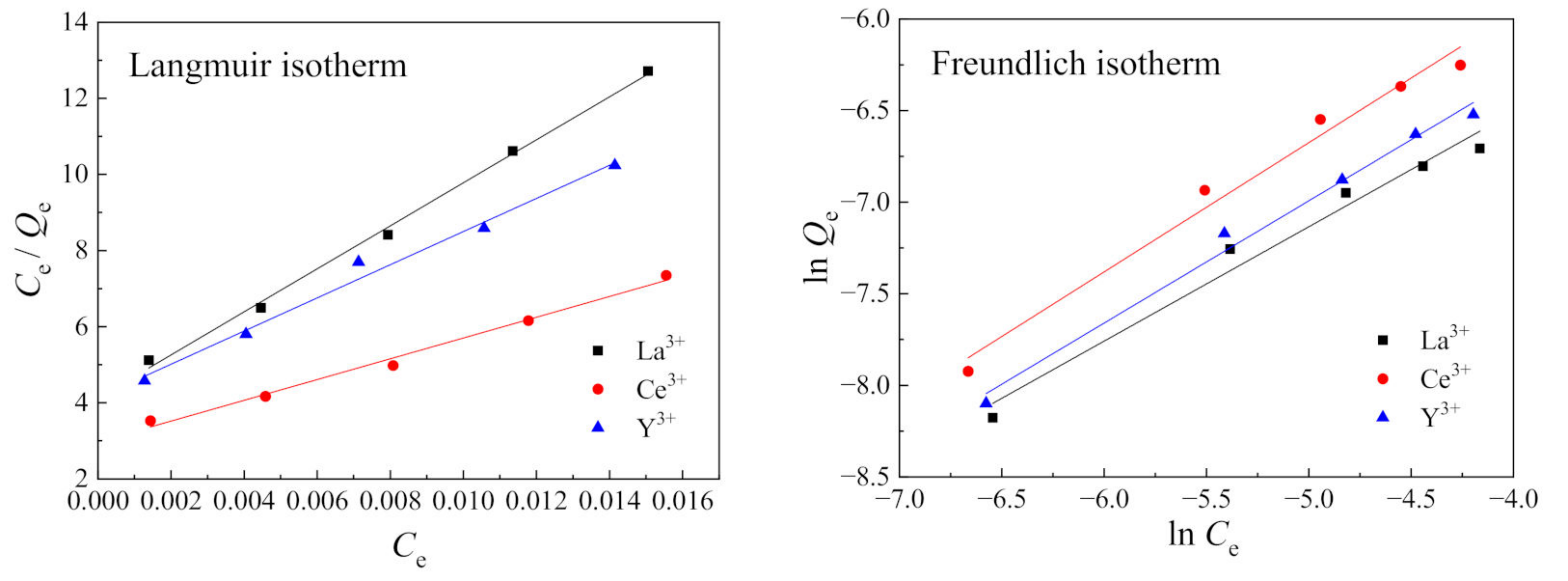
Linear forms of Langmuir isotherm and Freundlich isotherm.

Figure 9 reveals the linear plots of *C*_e_*/Q*_e_ vs. *C*_e_, ln*Q*_e_ vs. ln*C*_e_ and the calculated related parameters are listed in Table 7. The achieved results indicated that the Langmuir isotherm was more suitable to describe the adsorption of rare earth ions on PAMHA resin than the Freundlich. Compared with the Freundlich isotherm, the value of *R*^2^ from the Langmuir isotherm was closer to 1. Therefore, it can be concluded that the working adsorption system obeyed Langmuir isotherm and the adsorption process of the resin to rare metal ions might be monolayer adsorption.

### 3.7. Thermodynamics Adsorption

**Figure 10:**
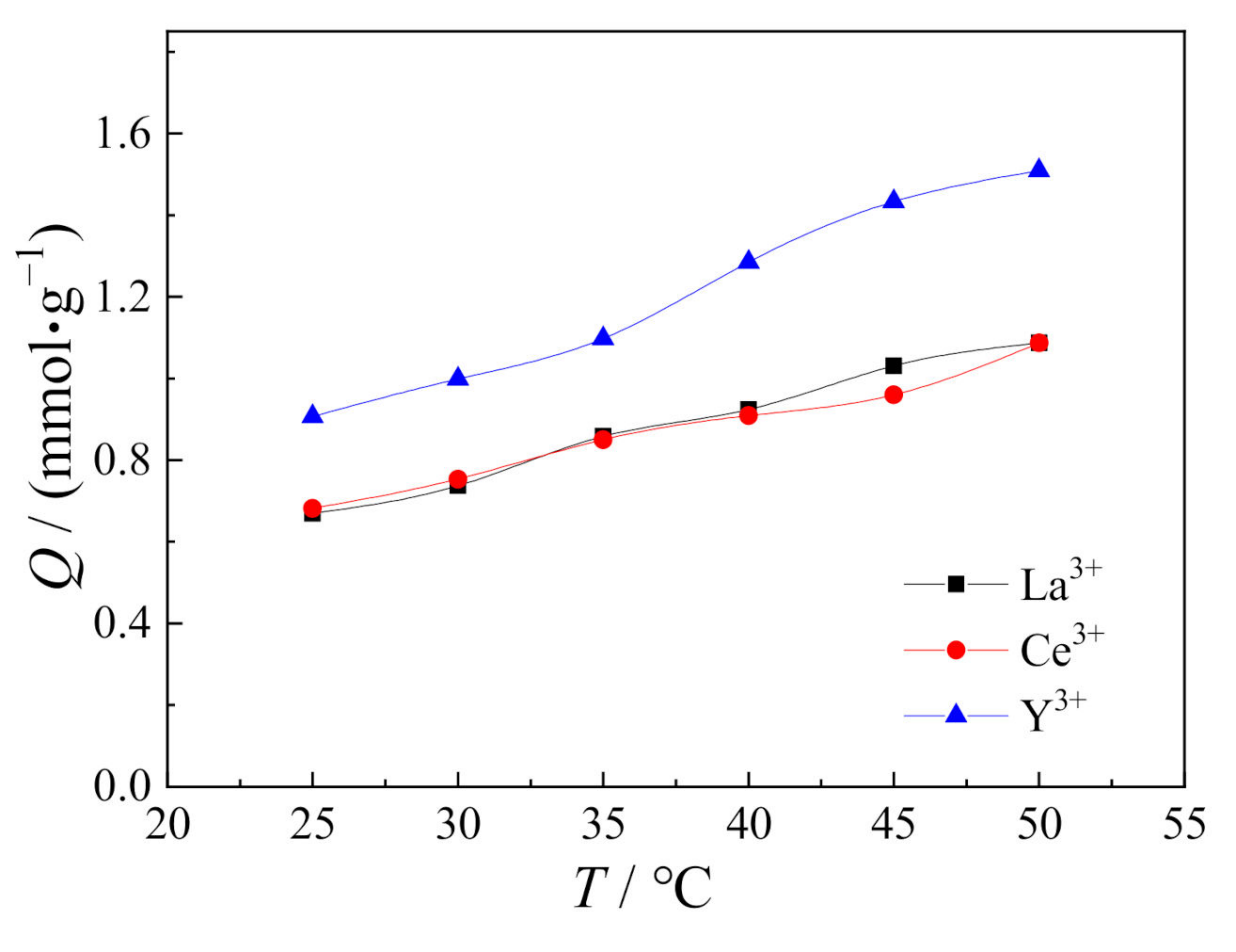
Effect of temperature on the adsorption capacity of PAMHA. (*C*_0_ = 0.01 mol·L^−1^, 5 h, pH values for La^3+^, Ce^3+^, and Y^3+^ were 1.5, 3.0, and 2.0, respectively.)

The effect of temperature on adsorption capacity of PAMHA resin for La(III), Ce(III), and Y(III) is displayed in Figure 10. It is clear that, for all the three metal ions, higher temperatures were more favorable for the increasing uptake of La(III), Ce(III), and Y(III) ions. So, it can be concluded that the adsorption of La(III), Ce(III), and Y(III) ions onto PAMHA resin was an endothermic process.

In order to better understand the spontaneous and heat exchange characteristics during adsorption, thermodynamic analysis is necessary. Thermodynamics parameters such as the changes of Gibbs free energy (Δ*G*), enthalpy (Δ*H*), and entropy (Δ*S*) play important roles in adsorption behavior and they can be calculated by the following Equations [46,47,48].

(8) $$D=\frac{V(C_{0}-C_{e})}{mC_{e}}$$

(9) $$\ln D=-\frac{\Delta H}{RT}+\frac{\Delta S}{R}$$

(10) ΔG=ΔH−TΔS

In these Equations, *D* (L·g^−1^) is the distribution coefficient, *V* (L) is the volume of solution, *m* (g) is the mass of the resin, *C*_0_ (mol·L^−1^) and *C*_e_ (mol·L^−1^) are the initial concentration and equilibrium concentration of La(III), Ce(III), and Y(III) ions, respectively, *R* (8.314 J·mol^−1^·K^−1^) is the gas constant, and *T* (K) stands for temperature.

**Figure 11:**
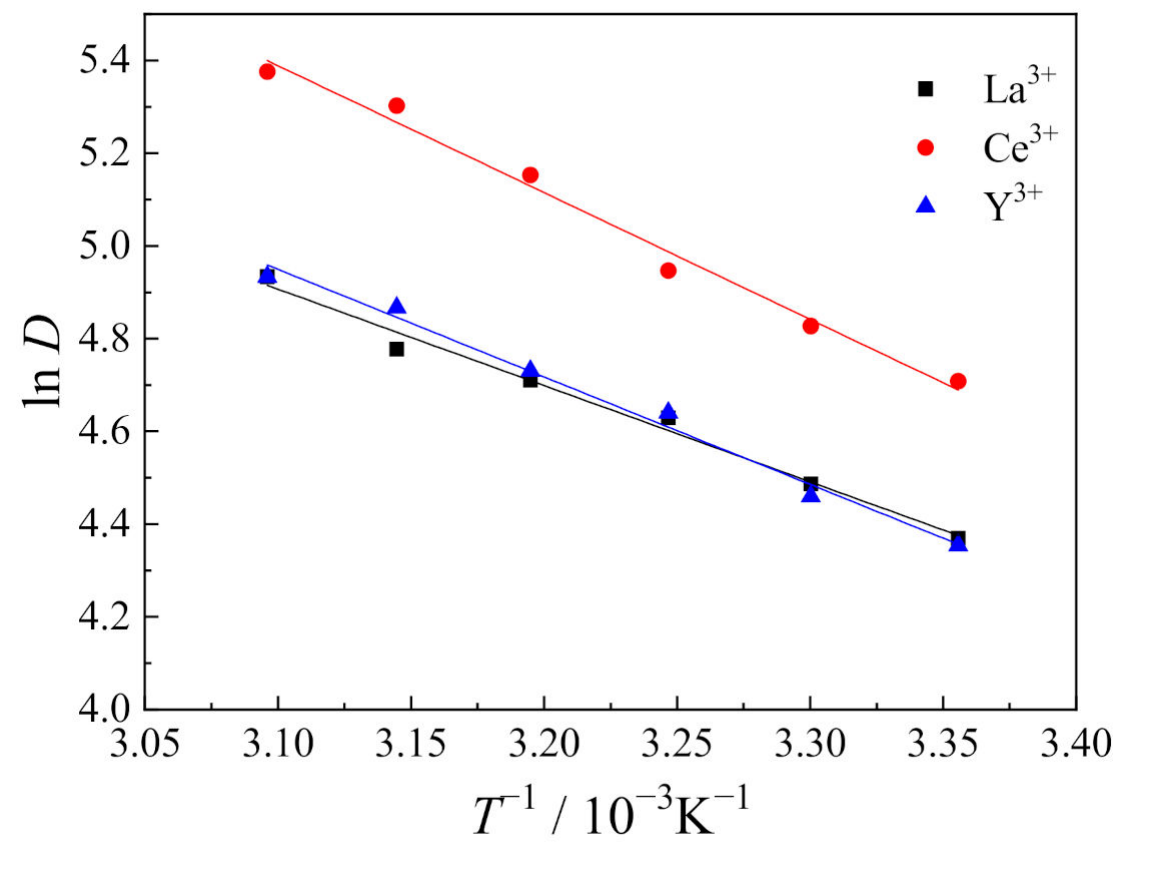
Linear fit plot of ln*D* vs. *T*^−1.^

The data from Figure 10 was fitted with the temperature coefficient method. The linear plot of ln*D* vs. *T*^−1^ is depicted in Figure 11 and the calculated thermodynamic parameters are shown in Table 8. Apparently, the minus Gibbs free energy values (Δ*G*) indicated the spontaneity of the adsorption system. The positive enthalpy values (Δ*H*) demonstrated that the adsorption process of La(III), Ce(III), and Y(III) ions on PAMHA resin was an endothermic reaction and the rising of temperature was beneficial to adsorption process. This confirmed the results of the effect of temperature on adsorption.

### 3.8. Desorption Experiments

**Figure 12:**
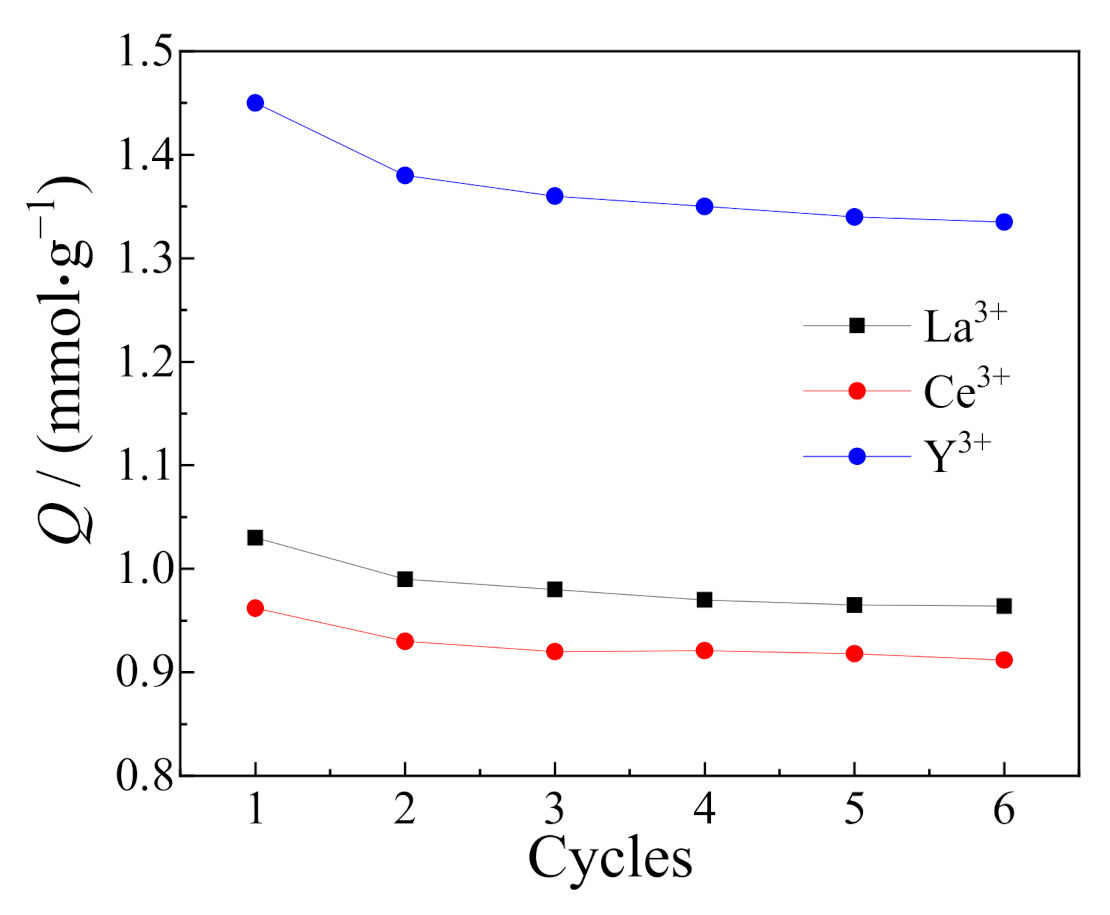
Regeneration of PAHMA.

The regeneration is an important factor to evaluate the potential of a newly-developed adsorbent. In this work, desorption tests were carried out using dilute nitric acid (2 mol·L^−1^) and rare earth ions can be effectively desorbed from PAMHA-complexes and desorption efficiencies for La(III), Ce(III), and Y(III) ions were 98.4, 99.1, and 98.8%, respectively. The adsorption–desorption operation was repeated for six cycles to investigate the reusability of PAMHA. As depicted in Figure 12, there was only a slight decrease of rare earth ions’ adsorption capacities after six cycles of regeneration operations (take Ce(III) as example—the adsorption capacity was only 5% decreased). The results indicated that PAMHA resin possessed good regeneration properties. Therefore, the obtained resin was possibly to be used as an absorbent for rare earth ions.

## 4. Conclusions

In the present study, we have first reported on a new resin containing both acylamino groups and hydroxamic acid functional groups and expected to have good coordination performance for rare earth ions. Batch adsorption results indicated that the resin possesses an excellent adsorption capacity for La(III), Ce(III), and Y(III) ions. SEM, FTIR, and XPS analyses showed that there was coordination between the rare earth ions with PAMHA and rare metal ions were chemically adsorbed on the surface of the PAMHA. The adsorption process followed pseudo-second-order model, indicating that chemisorption was controlled by the rate. The well fitted to Langmuir isotherm revealed that the adsorption was monolayer-dominated. Thermodynamic information showed that the adsorption of La(III), Ce(III), and Y(III) ions onto PAMHA resin was spontaneous and endothermic. Furthermore, six cycles of adsorption–desorption experiments suggest that PAMHA with high stability and reusability can be taken as a very promising and potentially attractive adsorbent for enrichment of rare earth ions.

## Figures and Tables

**Table 1:** Molecular energy, HOMO, and LUMO of acrylamide and acrylamide hexyl hydroxamic acid.

Molecule	Energy/(a.u.)	HOMO/(a.u.)	LUMO/(a.u.)
acrylamide	−247.307621563	−0.26592	−0.05158
acrylamide hexyl hydroxamic acid	−687.53675764	−0.26051	−0.05516

Note: HOMO—the highest occupied molecular orbital energy. LUMO—the lowest unoccupied molecular orbital energy.

**Table 2:** Net charges of selected atoms of acrylamide and acrylamide hexyl hydroxamic acid.

Atom	Charge	Atom	Charge
O1	−0.483	O6	−0.388
O2	−0.562	O7	−0.387
O3	−0.223	O8	−0.557
N4	−0.402	O9	−0.217
N5	−0.517	N10	−0.132
		N11	−0.520

**Table 3:** The functional groups correspond to the peaks in Fourier transform infrared spectroscopy (FTIR).

Wavenumber/cm^−1^				Functional Group
PAMHA	PAMHA-La(III)	PAMHA-Ce(III)	PAMHA-Y(III)	
3419	3440	3443	3446	N–H, O–H
2963	2913	2925	2923	C–H
1741	1701	1701	1707	C=O
1435	1456	1456	1460	–C=N
1158	1160	1155	1161	C–C

**Table 4:** Electronic binding energy of PAMHA before and after treatment.

Species	Binding Energy/eV					
	C 1s	O 1s	N 1s	La 3d	Ce 3d	Y 3d
PAMHA	284.82	531.98	400.87	—	—	—
PAMHA-La(III)	284.80	531.99	400.66	837.80	—	—
PAMHA-Ce(III)	284.80	532.01	400.12	—	885.67	—
PAMHA-Y(III)	284.80	532.03	400.32	—	—	158.32

**Table 5:** Adsorption capacity of PAMHA on rare earth ions.

Resin	Adsorption Capacity/(mmol·g^−1^)		
	La^3+^	Ce^3+^	Y^3+^
PAMHA	1.030	0.962	1.450
D113	0.681	0.778	0.442

**Table 6:** Kinetic parameters of different models.

Rare Earth Ions	Pseudo-First-Order			Pseudo-Second-Order			Elovich		
	*k*_1_	*Q*_1_	*R* ^2^	*k*_2_	*Q*_2_	*R* ^2^	*α*	*β*	*R* ^2^
La^3+^	0.7752	1.391	0.9732	0.1824	1.731	0.9886	1.226	2.710	0.9876
Ce^3+^	0.7796	1.245	0.9834	0.2453	1.515	0.9902	1.219	2.979	0.9891
Y^3+^	0.8284	1.798	0.9929	0.2371	2.077	0.9881	2.095	2.070	0.9887

**Table 7:** Langmuir and Freundlich adsorption isotherm parameters.

Adsorbate	Langmuir Isotherm			Freundlich Isotherm		
	*Q*_m_/(mmol·g^−1^)	*K*_L_/(L·mol^−1^)	*R* ^2^	*n*	*K*_F_	*R* ^2^
La^3+^	1.770	136.83	0.9960	1.602	0.0181	0.9675
Ce^3+^	3.664	91.89	0.9879	1.417	0.0431	0.9760
Y^3+^	2.295	105.24	0.9830	1.498	0.0259	0.9859

**Table 8:** Results of thermodynamic parameters.

Adsorbate	Fitting Equation	Δ*G/*(kJ·mol^−1^)	Δ*H/*(kJ·mol^−1^)	Δ*S/*(J·mol^−1^·K^−1^)
La^3+^	ln*D* = 11.3519 − 2.0791/*T*	−12.727	17.286	94.380
Ce^3+^	ln*D* = 13.8713 − 2.7363/*T*	−13.924	22.750	115.326
Y^3+^	ln*D* = 12.1424 − 2.3203/*T*	−12.812	19.291	100.952
